# Mediator Complex: A Pivotal Regulator of ABA Signaling Pathway and Abiotic Stress Response in Plants

**DOI:** 10.3390/ijms21207755

**Published:** 2020-10-20

**Authors:** Leelyn Chong, Pengcheng Guo, Yingfang Zhu

**Affiliations:** State Key Laboratory of Crop Stress Adaptation and Improvement, School of Life Sciences, Henan University, Kaifeng 475001, China; chong87@henu.edu.cn (L.C.); gpc0108@henu.edu.cn (P.G.)

**Keywords:** Mediator complex, transcription, ABA signaling, abiotic stress response

## Abstract

As an evolutionarily conserved multi-protein complex, the Mediator complex modulates the association between transcription factors and RNA polymerase II to precisely regulate gene transcription. Although numerous studies have shown the diverse functions of Mediator complex in plant development, flowering, hormone signaling, and biotic stress response, its roles in the Abscisic acid (ABA) signaling pathway and abiotic stress response remain largely unclear. It has been recognized that the phytohormone, ABA, plays a predominant role in regulating plant adaption to various abiotic stresses as ABA can trigger extensive changes in the transcriptome to help the plants respond to environmental stimuli. Over the past decade, the Mediator complex has been revealed to play key roles in not only regulating the ABA signaling transduction but also in the abiotic stress responses. In this review, we will summarize current knowledge of the Mediator complex in regulating the plants’ response to ABA as well as to the abiotic stresses of cold, drought and high salinity. We will particularly emphasize the involvement of multi-functional subunits of MED25, MED18, MED16, and CDK8 in response to ABA and environmental perturbation. Additionally, we will discuss potential research directions available for further deciphering the role of Mediator complex in regulating ABA and other abiotic stress responses.

## 1. Introduction

The Mediator is an evolutionarily conserved eukaryotic multi-protein complex that has been recognized as a key regulator of plant growth and development, plant defense, and hormone signaling transduction [1,2,3]. It regulates transcription through recruiting RNA polymerase II (Pol II) to specific gene promoters by linking transcription factors (TFs) bound at activators and repressors with the pre-initiation complex (PIC). Upon receiving and transferring regulatory signals to the basal transcriptional machinery, the Mediator complex undergoes conformational changes, which creates a flexible surface that aids the assembly of PIC. Functioning as a molecular bridge, the Mediator complex physically interacts with PIC as well as TFs to perform transcriptional activation [4,5,6]. Based on the classification from structural studies, the core Mediator is divided into the head, middle, and tail modules [7,8,9]. Each of these modules is made up of different subunits that characterize the distinct function of each module on transcription [10]. Depending on the species, the number of Mediator subunits may vary, and there are approximately 34 subunits reported in plant Mediator [11]. A number of Mediator subunits has already been revealed to have critical functions in various plant developmental processes, hormone signaling, plant defense, and abiotic stress tolerance [1,3,12,13]. The head module primarily associates with Pol II to affect transcription whereas the tail module is believed to play a highly significant role as it interacts with gene-specific TFs. The middle module is reported to be responsible for the transfer of transcription signal from the tail to the head, which may also interact with Pol II [14]. The fourth and separable kinase module, termed as the CDK8 module, which consists of CDK8, C-type cyclin (CycC), MED12, and MED13 subunits, has been indicated to exist in plants. *Arabidopsis* CDK8 was first reported to regulate floral organ identity [15]. It was later found to interact with MED14 and *Arabidopsis* LEUNIG, a transcription co-repressor [16]. Further studies on the *Arabidopsis* regulator of alternative oxidase 1 (*rao1*) mutant that carries a mutation in *CDK8* documented that *CDK8* regulates mitochondrial retrograde signaling under H_2_O_2_ and cold stress [17].

Due to the important role of Mediator complex in transcription, it is comprehensible to find a rise of studies revealing the engagement of Mediator complex in various responses to ABA and environmental disturbances such as biotic and abiotic stresses. The role of Mediator complex in response to biotic stresses has been well documented [1,3,18,19,20]. However, the function of Mediator in the context of ABA and abiotic stress response still require further investigation as only a few studies have examined the role of Mediator in responding to ABA as well as to environmental perturbation. Thus far, the Mediator complex has only been discovered to serve roles in cold, salt, and drought stresses. Despite these findings, more studies are still required to facilitate the research in this area. Therefore, this review will emphasize the regulatory roles of Mediator complex in the ABA signaling pathway, as it is a major phytohormone that contributes significantly to the plant’s ability in adapting abiotic stress. Furthermore, we will also discuss the most recently reported role of Mediator complex in three abiotic stresses of cold, high salinity, and drought.

## 2. The Importance of Mediator Complex in Transcriptional Regulation

The Mediator complex functions together with cofactors of Pol II to regulate gene expression at the transcriptional level. Mediator plays a significant role in assisting plants to adapt to environmental changes because TFs recruit the Mediator complex through protein–protein interaction to trigger the activation or repression of target genes in plants with the Pol II transcription complex [1]. A diverse range of biological processes in *Arabidopsis* is regulated by more than 1600 TFs [21]. TFs are linked with the Mediator complex since they are crucial components in the Pol II-based transcriptional machinery and the Mediator complex interacts with different TFs upon conformational changes or when environmental and cellular signals are perceived. More specifically, the subunits of the Mediator complex are the vital components that interact with the TFs to regulate transcription. In fact, 34 subunits of the Mediator complex that have been purified from *Arabidopsis* were reported thus far to have the possibility of interacting with different TFs [2]. In terms of ABA signaling and abiotic stresses, the Mediator’s role and regulation of gene(s) induced by each abiotic stress situation and ABA require further work in order to be fully understood. Hence, it is important to identify any potential interaction that may occur between Mediator subunits and TFs that are involved in the signaling pathway of each situation of the abiotic stress as well as in ABA signaling.

## 3. Mediator Complex as a Pivotal Regulator of ABA Signaling Pathway

ABA is a phytohormone that has profound functions in various developmental processes throughout the plant life cycle, such as seed germination and dormancy, organ size control, vegetative development, stomatal closure regulation, as well as senescence [22,23,24,25]. It has been reported that the concentrations of ABA can increase up to 50-fold under drought stress [26] and this is one of the most drastic changes observed thus far in the concentration of a plant hormone responding to an environmental stimulus. Due to ABA’s significant involvement in plants’ responses to various environmental stresses, the ABA signaling pathway has been studied extensively. Thus far, many of the key components of the pathway have been successfully identified [27]. Despite this, components in the downstream of ABA signaling pathway remain to be uncovered.

**Figure 1:**
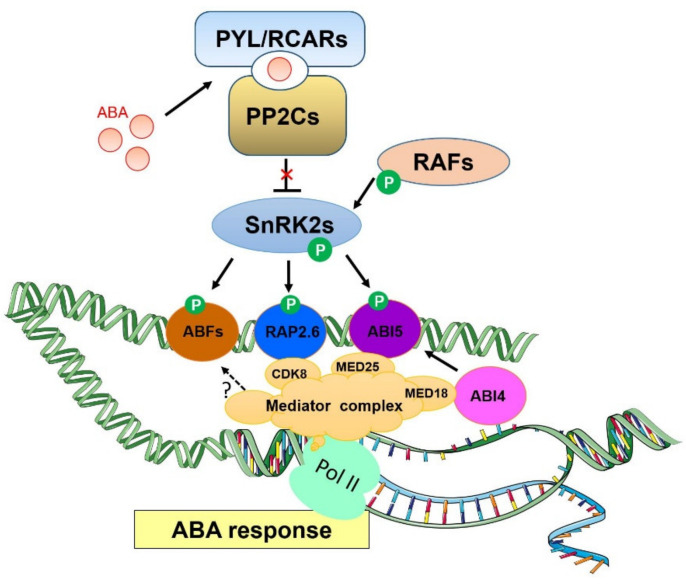
The pivotal role of Mediator complex in the ABA signaling pathway. ABA is perceived by its receptors PYL/RCARs, which promotes the interaction between PP2Cs (negative regulators of the ABA signaling pathway) and PYLs, hence releasing the positive regulators SnRK2s to activate ABA downstream signaling events. Additionally, RAFs can directly phosphorylate SnRK2s for the activation of SnRK2s, which subsequently interact with and phosphorylate several downstream TFs including ABFs, ABI5 and RAP2.6 to transduce the ABA signals. Mediator subunits of CDK8, MED25, and MED18 relay the signals from TFs RAP2.6, ABI5, and ABI4, respectively, and help recruit the RNA Pol II to the TFs-targeted promoters of ABA-responsive genes, thereby promoting the transcription of ABA-responsive genes.

Since 2009, a group of PYRABACTIN RESISTANCE (PYR)/PYR1-LIKE (PYL)/Regulatory Components of ABA Receptor (RCAR) proteins, members of a family of 14 START-domain-containing proteins in *Arabidopsis*, have been shown to function as the ABA receptors [28,29]. The core ABA signaling pathway also consists of the protein kinases in the SNF1-related protein kinase 2 (SnRK2) family, particularly SnRK2.2, SnRK2.3, and SnRK2.6/Open Stomata 1 (OST1) [27]. They have been shown to function as key positive regulators of ABA signaling [30,31,32,33]. Therefore, the earliest events occurred in ABA signaling require the presence of PYR/PYL/RCAR proteins, PP2Cs, and SnRK2 kinases (as shown in Figure 1). Without ABA, PP2Cs represses the kinase activity of SnRK2s as well as the downstream ABA signaling events. In the presence of ABA, it induces the formation of PYRs/PYLs/RCARs-ABA-PP2Cs complexes and PP2Cs will become inactivated, thereby permitting SnRK2s activation and the downstream events of ABA signaling [23,25,27,34]. The core ABA signaling pathway has been reconstituted successfully with those key components in vitro [35]. Interestingly, several recent studies simultaneously showed that Raf-like kinases (RAFs) could quickly activate SnRK2s to respond to ABA, osmotic and drought stress by direct phosphorylation [36,37,38,39].

The Mediator complex as described is a critical co-regulator of the transcriptional machinery and, unsurprisingly, it has also been found to serve important roles in the ABA signaling transduction. In fact, *MED25* is the first Mediator subunit that has been reported to act in response to ABA [40]. It was found that *MED25* negatively regulates the ABA signaling pathway as *med25* mutants display an increased sensitivity to ABA during seed germination and early seedling growth [40]. Consistent with its negative role in ABA signaling, *med25* mutant was noted to have an increased expression of ABA-responsive genes in response to ABA treatment compared to the wild type (WT) plants. ABA induced the transcription of *ABI5* (*ABA-INSENSITIVE5*), a key TF regulating the ABA signaling during seed germination [41,42,43], and, intriguingly, the ABA-induced transcription of *ABI5* was suppressed in *med25* mutants compared to WT. Nevertheless, ABI5 protein accumulated at higher abundance in *med25* mutants than that in WT, implying that MED25 may negatively regulate ABI5 at post-transcriptional level. Chromatin immunoprecipitation (ChIP) experiments further indicated that MED25 was highly enriched at the promoters of *ABI5* downstream genes, and this enrichment was reduced upon ABA treatment. MED25 was shown to directly interact with ABI5 and this interaction was attenuated by ABA, which was in accordance with the negative impacts of MED25 on the ABI5-regulated ABA responses. It is worth noting that *MED25* may be a critical regulator in hormones crosstalk between Jasmonic acid (JA), ethylene, and ABA signaling due to its interaction with MYC2 and several TFs in plants [44,45,46]. The head module subunit *MED18* has also been implicated in the ABA signaling. Opposite to *med25*, *med18* mutants are more insensitive to ABA at seed germination and early growth stages, similar to *abi4* and *abi5* mutants [47]. Remarkably, the induced expression of *ABI4* and *ABI5* by ABA are much lower in *med18* mutants than those in WT, indicating that the transcription of *ABI4* and *ABI5* are positively regulated by MED18. ChIP-qPCR revealed that MED18 is recruited to the ABI4 binding site on the *ABI5* promoter under both mock and ABA treatments. The physical interaction between MED18 and TF ABI4 further supports that MED18 regulates the ABA response and expression of *ABI5* through interacting with ABI4.

Recently, another subunit belonging to the Mediator kinase module termed as *CDK8*, has been identified as a critical regulator in the ABA signaling pathway [48]. As described previously, SnRK2s need to be phosphorylated by certain protein kinases in order to further perform the ABA signaling process [36]. CDK8 is known to possess kinase activity and this presents an opportunity for exploring its potential in regulating SnRK2s. Through utilizing genetic, transcriptomic, and biochemical approaches, CDK8 was solidified to associate with RAP2.6 and SnRK2.6 to positively regulate the transcription of ABA-responsive genes. *CDK8* mutation led to ABA insensitivity. Conversely, *CDK8* over-expression lines displayed hypersensitivity to ABA. Interestingly, the kinase-inactive version of CDK8 did not rescue the ABA phenotype of *cdk8* mutants, indicating the requirement of CDK8 kinase activity in the ABA response. The CDK8 and its kinase module components are generally known as negative regulators of gene expression in yeast, metazoan cells, and plants [13,49,50]. However, increasing evidence is showing that CDK8 could also play a positive role in plant transcriptional regulation as expression of defense-responsive genes (*PDF1.2*, *AACT1* and *NPR1*), salicylic acid (SA)-biosynthetic genes (*ICS1* and *EDS5*) and ABA-responsive genes such as *RAP2.6*, *RD29A*, *RD29B*, and *COR15A* [44,48,51,52] are positively regulated by *CDK8* in plants. Transcriptomic analysis has revealed that *CDK8* affects approximately 30% of the ABA-responsive genes, most of these genes are downregulated in *cdk8* mutants compared to WT. The expression of several important TFs (*DREB2A* and *RAP2.6*) and ABA-responsive genes (*RD29A*, *RD29B,* and *COR15A*) was found to be significantly lower in *cdk8* mutant plants. Therefore, this indicates a positive role of *CDK8* in modulating ABA-induced transcription. Moreover, ChIP analysis was utilized to verify that *CDK8* is essential for the ABA-induced Pol II recruitment to the promoters of ABA-responsive genes. In fact, RAP2.6, an ERF/AP2 type TF that involves in biotic and abiotic stress responses, was identified as a new interactor of CDK8 through a yeast two-hybrid screen. CDK8 was further shown to be enriched at the promoter region of *RAP2.6* in response to ABA, demonstrating that *CDK8* is an important component for regulating *RAP2.6* transcription. Moreover, *RAP2.6* was found to directly associate with the DRE or GCC motif and *RD29A* or *COR15A* promoters. In response to ABA, RAP2.6 could be enriched at the *RD29A* and *COR15A* promoters. These findings indicated the possibility that *CDK8* may regulate the expression of ABA-responsive genes through *RAP2.6* [48]. It may also be possible that other TFs interact with CDK8 to regulate the expression of ABA-responsive genes.

In addition, RAP2.6-mediated activation of *RD29A* has been observed to be attenuated in *cdk8* mutants, thereby showing that *CDK8* is required for the recruitment of Pol II to the promoters of RAP2.6 target genes. Consistent with biochemical results, the over-expression of *RAP2.6* resulted in hypersensitivity to ABA and mannitol as well as higher expressions of several ABA-responsive genes. These findings indicated that RAP2.6 and CDK8 could finetune the transcription of ABA-responsive genes, especially those genes containing DRE/GCC-motifs. Another important finding is that Mediator CDK8 could link the core ABA signaling component of SnRK2.6 to Pol II transcriptional machinery, which facilitates the immediate transcriptional response to ABA and abiotic stress. Although no direct interaction and phosphorylation between CDK8 and SnRK2.6 have been observed, it is possible that CDK8 associates with SnRK2.6 through RAP2.6 to form a ternary complex since both kinases directly interact with RAP2.6. In vitro kinase assays further indicated that RAP2.6 was phosphorylated by SnRK2.6, but not by CDK8. It therefore raises the possibility that RAP2.6 may act as a SnRK2.6 substrate or a downstream TF to transduce the ABA signaling, but it requires further genetic studies and in vivo phosphorylation evidence to support the existence of this ternary complex in plants. Future study should also elucidate whether the phosphorylation of RAP2.6 by SnRK2.6 could affect its transcriptional activity, protein stability or translocation. Although CDK8 did not directly phosphorylate RAP2.6 in vitro, the possibility of CDK8 kinase activity requiring either cyclin or other partners to promote its phosphorylation in vivo should not be excluded. Thus far, very few CDK8 substrates have been reported and this is likely due to its weak kinase activity in vitro. The pivotal roles of Mediator complex in the ABA signaling pathway are summarized in Figure 1.

## 4. Mediator Complex Is Vital for Plants to Respond to Abiotic Stresses

**Figure 2:**
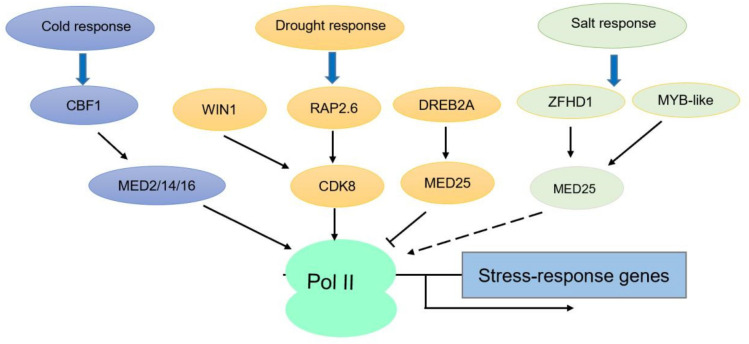
The simplified network of TFs and Mediator complex in regulating the abiotic stress responses. In response to cold stress, CBF1 activates the expression of *COR* genes through MED2, MED14, and MED16, which are required for the recruitment of RNA Pol II to the promoters of *COR* genes; In response to drought stress, CDK8 physically interact with WIN1 and RAP2.6 to positively regulate the cuticle wax biosynthesis and expression of stress-responsive genes; in contrast, MED25 negatively regulates the transcriptional activity of DREB2A and the expression of stress-responsive genes, thereby negatively contributing to the drought tolerance; in response to salt stress, ZFHD1 and MYB-like interact with MED25 to positively regulate the salt response.

In order to withstand disturbances in the natural environment, plants must be able to rapidly respond and adapt to environmental stimuli by dynamically changing the expression of genes that help them maintain cellular homeostasis. Abiotic stresses such as cold, high salinity, and drought are some of the environmental stimuli that plants get exposed to and they must be able to integrate these signals using different regulatory pathways if they are to survive [25,53]. Various subunits of the Mediator complex including CDK8, MED16, MED14, and MED25 have been identified to help plants to respond to these stresses [1,48]. We will summarize some of the findings that have been reported about the functions of these subunits in dealing with three abiotic stresses of cold, high salinity, and drought (Figure 2).

## 5. Mediator Subunits Modulate Freezing Tolerance in Plants

*MED16* is one of the first Mediator subunits that was reported to involve in abiotic stress response. *MED16* has been indicated to help plants overcome cold stress (freezing) through eliciting responses that maintain physiological metabolic homeostasis. Before MED16 was recognized as part of the Mediator complex, it was named as *SENSITIVE TO FREEZING6* (*SFR6*) and was identified for its role in cold acclimation-induced freezing tolerance [54,55]. The process of cold acclimatization involves the expression of many cold inducible/cold responsive or cold on-regulated (*COR*) genes such as *KIN1*, *COR15a*, and *RD29A* (specifically those consisting of C-repeat/dehydration-responsive element (CRT/DRE) elements in their promoter). The expression of *COR* genes is mainly induced by the TF of C-Repeat/DRE Binding Factor 1 (CBF1) [56]. In the study for its ability of freezing tolerance and cold acclimation, the *sfr6* mutants were observed to express significantly decreased levels of *COR* gene and protein accumulation and were thereby unable to tolerate freezing after cold acclimation. The CRT/DRE elements containing *COR* genes become uninducible at low temperature in *sfr6* mutants. It is very likely that SFR6/MED16 acts downstream of CBF1 and triggers the recruitment of Mediator complex to CBF1 responsive genes. To better understand the role of *MED16* in cold signaling, TFs *CBF1* and *CBF2* were also overexpressed in the *sfr6* mutant since CBFs are responsible for the activation of *COR* genes. It was found that the overexpression of *CBF1* and *CBF2* failed to increase the expression of *COR* target genes, further confirming that *MED16* acts downstream of CBF TFs [57]. In fact, *MED16* has been validated as an indispensable Mediator subunit in plants for activating CBF-regulated *COR* genes as, without MED16, Pol II is unable to be recruited to these genes [58]. The plant’s ability to survive cold stress relies on the effective induction of *COR* genes and interestingly, without *MED16*, *COR* genes are unable to be induced, and this further causes osmotic stress sensitivity in *sfr6* mutants [55]. The *med16* mutant was also reported for its hypersensitivity to iron deficiency and sensitivity to excessive zinc, which could be rescued by increasing iron concentration. Additionally, MED16 was proven to interact with MED25 to regulate iron homeostasis [59]. Despite its association with MED16 to regulate biological processes, *MED25* was not involved in cold acclimation-induced freezing tolerance.

In addition to *MED16*, *MED14* and *MED2* are two additional Mediator subunits that have been shown to have an effect on *COR* gene expression, further signifying the importance of Mediator in plant’s adaptation to cold stress [58]. More importantly, all three tail module subunits of MED16, MED14, and MED2 play a significant role in recruiting the Pol II to the CBF1 target genes to regulate cold stress response [58], suggesting the essential role of Mediator complex in the cold response.

## 6. Multi-Functional Roles of Mediator in Salt and Drought Stresses

Salt and drought are two major abiotic stresses that limit crop yield worldwide. The SOS (Salt Overly Sensitive) signaling pathway is extensively reported to contribute to salt tolerance in plants [53,60]. The transcription of TFs is also essential for the salt and drought response in plants [61]. Thus far, only *med25* and *med18* mutants have been reported to exhibit a reduced tolerance to salt stress [45,62]. MED25 was found to interact with several TFs of DREB2A (drought response element protein B), ZFHD1 (zinc finger homeodomain 1) and MYB-like from yeast two-hybrid screen using the conserved activator-interacting domain (ACID) of MED25 as a bait. Consistently, mutation in *MED25*, *DREB2A*, *ZFHD1*, and *MYB-like* all caused an increased sensitivity to salt stress [45]. Nevertheless, the salt-responsive genes that are affected by *MED25* and those TFs are not reported. MED18 was found to interact with NUP85 and positively contribute to the ABA signaling and salt tolerance [62]. A recent work also reported that four Mediator subunits (*MED9*, *MED16*, *MED18*, and *CDK8*), representing four different modules, are required for salt stress and thermal stress mediated transcriptional responses by RNA-seq analysis in *Arabidopsis* [63]. However, limited studies have been reported on the roles of Mediator complex in salt stress responses. The detailed mechanism of how Mediator subunits regulate the salt response remains unclear. It is unknown if Mediator complex could affect the SOS pathways and any other critical transporters.

Besides salt stress, *MED25* is also involved in drought stress. The *med25* mutant has been indicated to display an increased resistance to drought, as opposed to its salt sensitivity [45]. MED25 is involved in modulating drought stress response through interacting with DREB2A. DREB2A consists of both repressing domain (RD) and activating domain (AD) in its protein sequence [64,65]. The mutation of *dreb2a* and *med25* has been demonstrated to have an opposite effect in drought stress as *dreb2a* was found to exhibit drought sensitivity while *med25* displayed an increased resistance to drought. The explanation provided for the observed opposite effect of *MED25* and *DREB2A* in drought stress was that MED25 acts as the corepressor of DREB2A in drought stress by interacting with the AD in DREB2A and depositing some other Mediator subunit in close vicinity of DREB2A RD. Therefore, when *MED25* is disrupted, the repressor function is lost and DREB2A activates genes involved in drought [45]. Based on the evidence presented about *MED25*, it appears that *MED25* mainly plays negative roles in abiotic stress response.

In addition, *CDK8* has also been indicated to participate in drought stress recently. *CDK8* mutation results in higher stomatal density and impaired stomatal aperture, as well as reduced tolerance to drought [48]. Consistently, over-expression of *CDK8* enhances the drought tolerance. Considering the enhanced cuticle permeability and thinner cutin observed in *cdk8* mutants [44], it is likely that *CDK8* regulates the drought response through multiple mechanisms. Remarkably, CDK8 was found to directly interact with ERF/AP2 type TFs WIN1 (WAX INDUCER1) and RAP2.6, which are key regulators of cuticle wax biosynthesis and an abiotic stress responsive gene, respectively [44,66]. CDK8 positively regulates cutin biosynthesis and wax accumulation through interacting with WIN1. Interestingly, in addition to playing a role in the wax biosynthesis, WIN1 also participates in abiotic stress response as its expression is significantly induced by various abiotic stresses and WIN1 can also bind the GCC-box and DRE element sequences to activate several stress-responsive genes [67,68], implying the potential function of CDK8–WIN1 interaction in the drought response. Furthermore, CDK8 also contributes to drought tolerance by cooperating with RAP2.6-SnRK2.6 complex, which could facilitate the immediate transcription of stress-responsive genes. Therefore, Mediator subunits are capable of different functions and can perform different roles depending on the type of environmental stress.

## 7. Conclusion and Perspectives

In response to abiotic stress, plants must appropriately regulate gene expression in a synchronized manner. It is unsurprising to find that the Mediator complex is linked with the ABA signaling pathway and abiotic stress as it has important roles in transcriptional regulation. Despite the confirmed relationship of Mediator complex with ABA and abiotic stress response, the molecular mechanism of Mediator complex in regulating the ABA and abiotic stress response remains elusive. Thus far, only a few Mediator subunits have been reported to be involved in the ABA signaling pathway, cold (freezing), salt, and drought response. Since more than 30 Mediator subunits have been documented in plants, this presents an opportunity for discovering if there are more subunits involving in the ABA signaling and abiotic stress response in future. Furthermore, the plausible roles of Mediator complex in heat stress and submergence are worthy of an investigation as knowledge about the mechanism of heat and submergence stress response is still limited. Therefore, it is desirable to screen all the Mediator subunits and identify the ones that exhibit functions in the ABA signaling pathway as well as in abiotic stress that has not yet been fully studied.

As revealed from structural studies, the Mediator complex is divided into four distinct modules and it is still unclear whether each module could exert specific effects on the ABA signaling transduction or abiotic stress response in plants. Future studies should address whether the subunits within the same module present overlapping or opposite roles in regulating the ABA and abiotic stress responses. It is known that *med25* mutants are sensitive to ABA, while the *cdk8* and *med18* mutants are even less sensitive to ABA. It is necessary to study the detailed mechanism of how those Mediator subunits coordinately or completely regulate the ABA or abiotic stress. Given the nature that Mediator complex functions between Pol II and TFs, it is also necessary to identify additional TFs that interact with different Mediator subunits in response to ABA and abiotic stress. Currently, only a few TFs (DREB2A, ABI5 and RAP2.6, etc.) have been reported to interact with Mediator subunits to regulate ABA and abiotic stress responses. Undoubtedly, this area is drawing the attention of plant scientists as the Mediator complex profoundly participates in transcriptional regulation. High-throughput proteomics and protein–protein interaction approaches are essential for improving the knowledge of this field and they should be further improvised to uncover more TFs that directly interact with specific Mediator subunits, which will then provide deep insights into the molecular mechanism of Mediator complex in the regulation of ABA signaling and abiotic stress. Furthermore, it would be interesting to find if there are potential ABA or stress-induced dynamic interactions between MED and TFs in response to a specific environmental stimulus.
